# Co-design of health educational materials with people experiencing homelessness and support workers: a scoping review

**DOI:** 10.3389/froh.2024.1355349

**Published:** 2024-06-11

**Authors:** Andrea Rodriguez, Shambhunath Shambhunath, Thushani Indumani Devi Wijesiri, Camila Biazus-Dalcin, Niall Mc Goldrick

**Affiliations:** ^1^ Dental Public Health, School of Dentistry, University of Dundee, Dundee, United Kingdom; ^2^ Mother, Infant and Child Research Group (MIRU), School of Health Sciences, University of Dundee, Dundee, United Kingdom

**Keywords:** homelessness, co-design, oral health, health, health promotion, training, education

## Abstract

**Introduction:**

People experiencing homelessness are often marginalised and encounter structural barriers when seeking healthcare. Community-based oral health interventions highlighted the need of well-trained practitioners for the successful engagement of service users and behaviour change. However, a lack of adequate information and specific training has been previously reported. The adoption of inclusive approaches, such as co-design, to develop tailored and meaningful health promotion training and educational materials capable of addressing the specific needs of this group is required. Co-design entails active involvement of different groups in research processes that acknowledge participants' needs and expectations. This scoping review aims to identify the available literature on the participation of people experiencing homelessness and/or their support workers in co-designing health and oral health promotion training/educational materials, approaches adopted, and barriers and enablers to develop these materials.

**Methods:**

The Joanna Briggs Institute (JBI) Scoping Review Methodology informed the development of the scoping review. The protocol was registered on the Open Science Framework. Six electronic databases (Medline (OVID), PsychInfo (OVID), Scopus, Web of Science, Applied Social Sciences Index and Abstracts (ASSIA) (ProQuest) and CINHAL) were systematically searched using MeSH terms. An extensive grey literature search, consultation with experts and hand searching of reference lists took place. Records were screened independently and in duplicate using the Rayyan Qatar Computing Research Institute (QCRI) online tool, followed by qualitative content analysis involving descriptive data coding.

**Results:**

Eight studies/materials were included. Key approaches adopted to co-design, enablers and barriers were captured. The enablers were inclusivity, a safe environment for positive participation, empowerment and flexibility, the barriers were difficulty in recruiting and sustaining participation, power differentials, and limited resources.

**Conclusion:**

The evidence in this area is limited. This scoping review provided foundations for further research to examine the impact of different components of the co-design process including the environment in which the co-design process is conducted. Further studies with experimental design and reported using appropriate study design frameworks detailing active components of the co-design process would strengthen the evidence base in this area.

## 1 Introduction

People experiencing homelessness are socially excluded and face structural barriers to accessing healthcare, leading to high physical and psychosocial morbidity and mortality (1). In the UK, the definition of homelessness extends beyond the mere absence of shelter, and instead encompasses a range of interconnected aspects such as experience of extreme poverty, domestic violence, job loss, and inability to afford rent (2, 3). As a result, individuals who are experiencing homelessness face a myriad of interconnected challenges stemming from their diverse and complex health and social needs (4). These intricate physiological, socio-economic, and psychological issues require joint multi-sector efforts to fully comprehend and tackle (5). Gaining a better understanding of the context and social determinants of health that may be affecting individuals experiencing homelessness is crucial for practitioners, in order that practitioners feel equipped to embrace a more inclusive approaches that will engage this population, ensuring their continued involvement in health care interventions (6, 7). Previous research about community-based oral health interventions has confirmed that well-trained and motivated practitioners are a key component that leads to engagement of service users and subsequent behaviour change (6, 8).

Whilst it is crucial for practitioners to establish trust with marginalised populations, a lack of adequate information and/or specific training to aid with this has been reported (7). Therefore, improved training and educational resources could help practitioners to engage, build trust and therefore discuss a broader range of sensitive health topics (9). Alongside this, people with lived experience of homelessness have expressed that they could be listened to more and be better supported when accessing services (10).

Therefore it is vital to involve people with lived experience of homelessness and their support workers in the development of health educational and health promotional materials and interventions, to ensure the resources are meaningful and acceptable (11). It has been found that involving people with lived experience can lead to effective strategies to address health needs and improve policies to tackle health inequalities (12, 13). The World Health Organization (WHO) has recently launched a framework to support meaningful engagement with a view to enhancing policies and services (14). The framework includes principles such as power, equity, inclusivity, contextualisation, elimination of stigmatisation, and institutionalisation of engagement (14).

Co-design is a participatory approach that brings individuals together to collaborate and combine their knowledge, skills, and resources to accomplish a design task (15). Co-design transcends mere consultation, originating from participatory design (15), it involves the meaningful engagement of end-users who are recognised as experts by experience (16). This approach is particularly powerful for socially excluded groups, empowering individuals by acknowledging their views and experiences (11). Furthermore, co-design serves as a pivotal approach for tackling stigmatisation and promoting inclusivity, the creation of co-designed materials counteracts societal stigmatisation (17). Co-design techniques have been reported to result in increased applicability and acceptance of research questions, outputs, participants' engagement, increased knowledge of different contexts, and an improved community network for the researcher (18).

Hence, it is imperative to scrutinize existing literature regarding the involvement of individuals who are homeless and/or their support workers in the creation of health and/or oral health educational materials through a co-design methodology, to elicit evidence to support best practice. Prior to conducting this review, a search of the literature for existing reviews of any type found no evidence synthesis addressing our aim. In the absence of any review, a scoping review methodology was chosen to scope the literature and identify evidence gaps.

To accomplish the main aim, three specific objectives were outlined:

(1) To summarise the literature in the field of co-designed health and/or oral health promotion training/educational resources that involved people experiencing homelessness and/or their support workers.
(2) To identify co-design approaches used in the development of training/educational materials such as health promotion guides, toolkits, workshop, and training programmes.
(3) To explore barriers and enablers to co-design health and/or oral health training/educational materials.

## 2 Methods

This scoping review was undertaken following the methodology established by the Joanna Briggs Institute (JBI) (19). An initial search in April 2021 of Scopus, PROSPERO (International prospective register of systematic reviews) and Open Science Framework (OSF) found no existing scoping or systematic reviews on this topic. A protocol for this scoping review was registered within the OSF database *a priori* (number osf.io/7hbac). Due to lack of research team capacity in 2021 and impact of the COVID-19 pandemic the search for the included literature in our review was last done in August 2023. A scoping review is an essential first step to inform future studies related to co-design of health promotion materials for people experiencing homelessness.

The reporting of this review aligns with the PRISMA extension for Scoping Reviews—PRISMA-ScR, we used population, concept and context to develop the review question and the eligibility criteria (20).

• Population: People experiencing or at risk of experiencing homelessness and/or support workers that work with people experiencing homelessness.
• Concept: Co-design approaches to produce health and/or oral health promotion training/education materials.
• Context: All settings and period considered.

This review outlines co-designed health and/or oral health promotion training/educational resources that involve people experiencing homelessness and/or their support workers. The research question was: (1). What is the range and nature of the existing empirical and non-empirical research using co-design approaches involving people experiencing homelessness and/or their support workers, to produce health and/or oral health promotion training/educational resources?

### 2.1 Search strategy

The search strategy was developed with the support of a Librarian, using specific Mesh terms and keywords (Supplementary Appendix S1), representing four broad themes: homelessness, health, oral health, co-design, and education and training material (Table 1).

**Table 1:** Eligibility criteria.

Inclusion Criteria	Rationale
Article in the English language	The dominance of English in academic research allowed wide access to pertinent information, yet limitations in resources and time, restricted searches in other languages.
All periods	There is no rationale to exclude any search period because the aim is to explore all existing literature on the topic.
Studies/materials need to address the development of health and/or oral health promotion co-designed training/ educational materials. Co-design was not specifically defined, as it was likely that there would be variance in the terms used in the global literature.	The studies/materials focus on health and/or oral health promotion addressing on health equity and social justice agenda.
Studies/materials with a population of people experiencing or at risk of experiencing homelessness and/or their support workers	The studies/materials involved people with lived experience and support workers who work with this population to develop relevant training/ educational material.
Exclusion Criteria	Rationale
Studies/materials involving participants younger than 16 years old.	It is not the target population of the study.
Studies/materials that do not follow the co-design process	The research/materials focus is the meaningful involvement of end-users in developing materials, which is more than consultation.
Reviews of the literature	The focus of the research is on the experiences from empirical studies.

**Figure 1:**
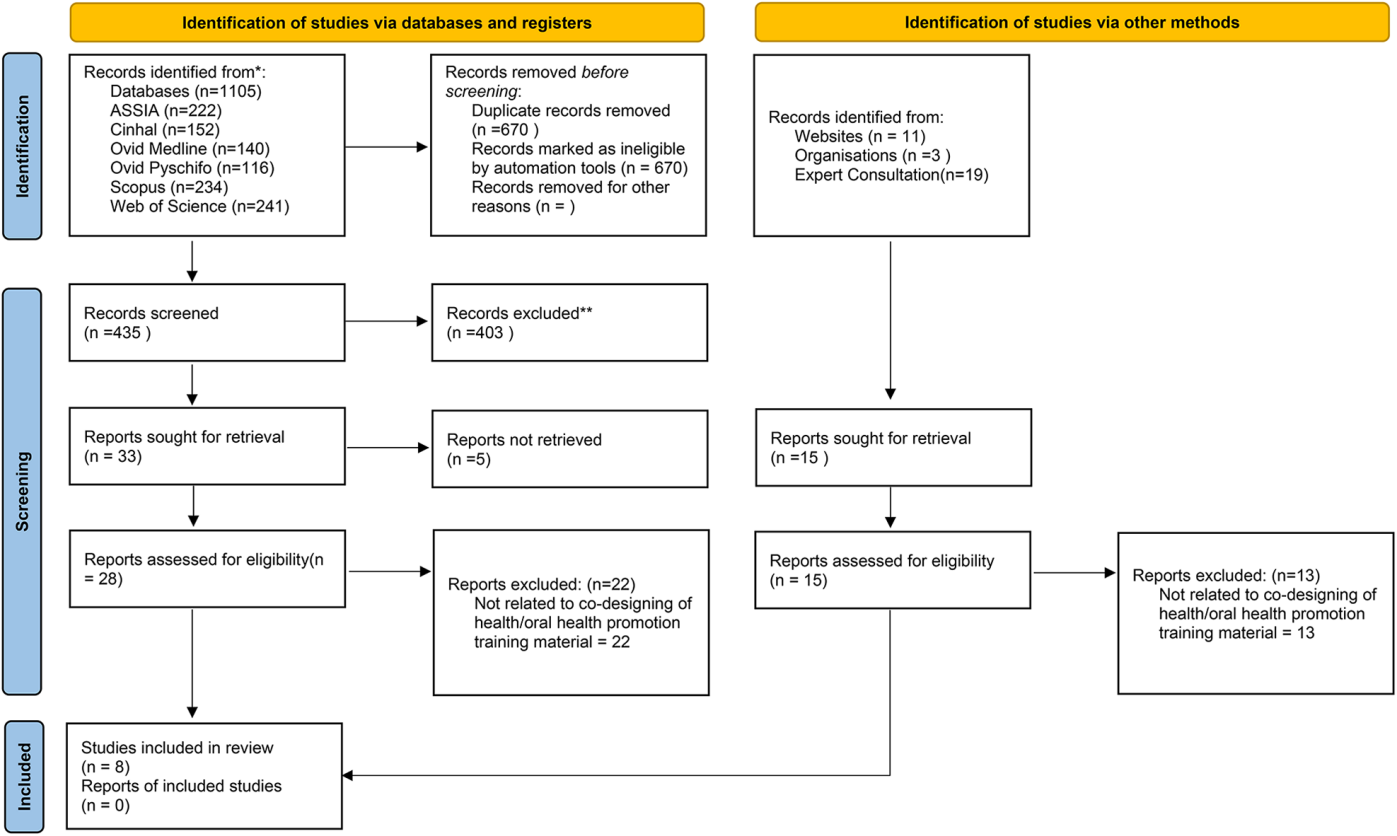
Review profile.

The literature searches were conducted in six electronic databases: Medline (OVID), PsychInfo (OVID), Scopus, Web of Science, Applied Social Sciences Index and Abstracts (ASSIA) (ProQuest) and CINHAL. In addition to database searches, supplementary search methods were employed including hand-searching reference lists of included studies, a grey literature search such as conference papers, reports, guides, toolkits, manuals, and website information using the Google Scholar-Advanced Search tool (Figure 1). Further, the authors contacted a range of international experts/stakeholders in this field to elicit further published materials. A grey literature search and contact with experts/stakeholders was deemed essential by the authors to ensure no relevant materials were missed and to comply with JBI Scoping Review guidance. Any published literature, such as papers published in peer-review journals, guidance documents, tool kits, knowledge exchange packages, reports, websites, and book chapters were in scope. Study methodology or quality did not impact decisions to include material. Any study design (including qualitative, quantitative and mix-methods studies) was within the scope.

#### 2.1.1 Contact with relevant stakeholders and experts in the field

This component provided unique feedback from group of stakeholders into the literature. The research team approached nineteen stakeholders (such as people with lived experience in homelessness, health practitioners, health educators, WHO officers, policymakers, and senior academics) by email or videocall to identify any further material that could meet the eligibility criteria.

#### 2.1.2 Data selection

Following the electronic database search (final search August 2023), articles that met the eligibility criteria were stored in EndNote, and any duplicate copies were removed manually (SS). The finalised list was imported to Rayyan Qatar Computing Research Institute (QCRI) (21), where titles and abstracts were screened blind and in duplicate (SS and TW)Any conflicts were resolved through discussion with an additional reviewer (NM). Subsequently, at least two reviewers (SS, TW, AR, CBD) independently read the full text of the eligible studies to confirm the inclusion of the studies in the review. Discussion took place with a third reviewer to resolve any conflicts. The PRISMA-ScR (Figure 1) demonstrates flow of papers in this review. Inclusion and exclusion criteria are presented in Table 1.

### 2.2 Quality assessment, data extraction and data synthesis

Although quality assessment is not a mandatory step in scoping reviews, we elected to undertake an assessment of the quality of the published studies included in this review to enhance utility of the output from our review and provide a view on the overall quality of research in this field. To maintain objectivity for those included studies where members of the review team were authors, an alternative team member assessed quality. The quality was assessed using the relevant JBI Critical Appraisal Checklist for Qualitative Research (22) and the MMAT Mixed Methods Appraisal Tool (23) dependent on study design. The quality report results (Supplementary Appendix S1). General database search terms were not used to determine inclusion in the review. After screening the included studies for quality two studies were considered high (9, 24) two studies were considered medium (25, 26) and one study was considered low (27).

The data extraction form was adapted from JBI (19) and was carried out independently by two researchers (SS and TW). The information extracted was title, authors, year of publication, journal of publication, type of publication, country of origin, aim, study sample, methodology, co-design approach, type of training/educational material developed, training aims, summary of key findings, and recommendations. Thematic analysis (28) was undertaken to construct themes from the included literature using the study objectives as a framework.

## 3 Results

A total of 1,105 papers were retrieved in the electronic literature search, and after the removal of duplicates, they were reduced to 435. Following title and abstract screening, twenty-eight papers were included for full-text screening. Twenty-two were excluded after full text screening, resulting in the inclusion of five papers (Figure 1). Two further resources were found via a grey literature search (*n* = 1) and the contact with experts/stakeholders (*n* = 1).

### 3.1 Study characteristics

In total eight papers/resources were included: five journal articles (9, 24–27) a conference paper (29), a training resource (30) and a workshop guide (31). All the papers/resources were published from 2018 to 2022, with five from the UK (9, 27, 29–31) two from Australia (24, 25) and one from Sweden (26). A summary of key characteristics of included evidence is presented in Table 2.

**Table 2:** Study characteristics.

Title	“No-one has listened to anything I’ve got to say before”: Co-design with people who are sleeping rough	Strengthening Social Interactions and Constructing New Oral Health and Health Knowledge: The Co-design, Implementation, and Evaluation of A Pedagogical Workshop Program with and for Homeless Young People	Co-designing a training package to promote health/oral health for people experiencing homelessness	Smile4life A co-designed educational and training resource	Technology for societal change: Evaluating a mobile app addressing the emotional needs of people experiencing homelessness	Sexual and reproductive health and rights (SRHR) education with homeless people in Sweden	Corrigendum to “The My Strengths Training for Life™ program: Rationale, logic model, and description of a strengths-based intervention for young people experiencing homelessness”	Do Not Give Up On Us. A workshop guide for health promotion and civic engagement
Authors	R M Mullins, B E Kelly, P S Chiappalone, V J Lewis	A Rodriguez, L Beaton, R Freeman	A Rodriguez, C Biazus-Dalcin, N McGoldrick, L van Blerk, C Murray, R Freeman	A Rodriguez, C Biazus-Dalcin, J Marshall, R Gorman	R Burrows, A Mendoza, S Pedell, L Sterling, T Miller, A Lopez-Lorca	E Wikström, E M Eriksson & M Lindroth	J Cumming, R Whiting, B. J. Parry, F. J. Clarke, M. J.G. Holland, S. J. Cooley, M. L. Quinton	A Rodriguez, C Biazus-Dalcin, L van Blerk
Year of publication	2021	2019	2021	2022	2022	2018	2022	2022
Journal/place of publication	Health Expectations	Dentistry journal	14th European Public Health Conference 2021	Discovery Research Portal—University of Dundee, UK	Health Informatics Journal	Sex Education	Evaluation and Program Planning	Discovery Research Portal—University of Dundee, UK
Type of study/material produced	Journal Article	Journal Article	Conference paper	A co-designed educational and training resource	Journal article	Journal Article	Journal Article	A workshop guide
Country of origin	Australia	Scotland, UK	Scotland, UK	Scotland, UK	Australia	Sweden	UK	Scotland, UK
Aim	To describe and evaluate a co-design project involving people with experience of rough sleeping to identify health, social and legal issues faced when sleeping rough.	To use critical consciousness as an educative tool to co-design, implement, and evaluate a series of oral health and health pedagogical workshops to strengthen social engagement and to construct new health knowledge with and for homeless young people and their service providers.	To co-produce training resources to support front-line staff in discussing and promoting health and oral health for people living with homelessness.	To enable practitioners and support workers from different backgrounds to provide evidence-based, tailored oral health promotion sessions through meaningful conversations with their service users	To the design and evaluation of the web app Ask Izzy	To describe and critically reflect upon the implementation of the Snacka Sex educational programme	To describe a multi-faceted strengths-based psychoeducational intervention for improving wellbeing and social inclusion of young people experiencing homelessness or at risk.	To provide structured activities and suggested resources to be explored in eight workshops topics on health promotion and civic engagement by third and health sector practitioners interested in improving engagement, health knowledge and the participation of young people they interact with.
Study Sample	81 people with recent rough sleeping experiences. A twelve-member working group, including seven men, four women, and one non-binary person, with diverse backgrounds: four born overseas, two culturally diverse, 4 LGBTQI, and one Indigenous.	Thirteen young homeless individuals (8 females, 5 males, aged 18–22) and five NGO staff (2 males, 3 females).	People with lived experience of homelessness, practitioners and students from the health and third sector, and policymakers	N/Applicable	30 participants (14 with current and lived experience of homelessness; 15 service providers, one software company)	85 participants (40 staff members, 45)	6 focus groups with 15 young people (10 male, 5 female; all current residents of the Service) and 18 frontline staff (6 male, 12 female).	N/Applicable
Co-design approach	A background survey followed by a working group (WG) with 12 weekly meetings. Interviews with participants of the WG. The analysis followed a deductive approach aligned with co-design principles (inclusion, equity, capacity building, and a purposeful approach).	The workshop program development was guided by Paulo Freire's principles (Dialogical Approach, Critical Consciousness, Action for Change) and involving three phases with the goal of encouraging participant reflection and co-creation of strategies for positive and healthy life changes. The program's evaluation was measured through direct observation, recorded workshops, post-workshop in-depth interviews, and post-workshop questionnaires. Content analysis was employed.	Community-based participatory research that used online workshops	The guide was co-designed through interviews and workshops representatives of with seven organizations (from health and third sectors) and individuals with homelessness experience.	Emotion- Led approach with a Living Labs process to design the web app, bringing together the different perspectives and capabilities from academia, industry, government, and citizens. Involved the following phases: discovery phase, research phase, vision phase, initial design and prototyping phase, design validation phase, and evaluation of the effectiveness of the app (semi-structured interviews conducted six months after the launch of the App).	The study had four phases (preparation, creation, realization, and evaluation) Both staff and service users were engaged in shaping the SRHR education by gathering their input, feedback, and experiences through surveys, staff meetings, field notes, and group sessions.	The intervention used Community-Based Participatory Research (CBPR) principles. It was iteratively developed through action research cycles, including a literature review, focus groups with young people and staff, and an initial pilot work with 15 participants. Feasibility was assessed through face-to-face community-based sessions and an outdoor residential course.	This workshop guide involved active collaboration from young people and third sector practitioners that attended eight workshop sessions to provide key elements/content added into the resource.
Methodology	Mixed methods	Qualitative Research	Qualitative Research	N/Applicable	Mixed methods	Mixed methods	Qualitative Research	N/Applicable
Type of training/educational material developed	An informal magazine: Zine A website: (http://www.needtoknowhomeless.org.au/). A dissemination event (information, stories, encouragement and advice for individuals experiencing homelessness).	Pedagogical workshop programme on health promotion and social participation.	Educational materials (e-book on health promotion, comics books on barriers to access services, and a guide to promoting oral health) compose this training package.	A co-designed educational and training guide on oral health for those working with people experiencing homelessness.	Web app “Ask Izzy”.	“Snacka” Sex educational programme	The My Strengths Training for Life™ program—MST4Life	A workshop guide on health promotion and civic engagement.
Training aims	To address health, social, and legal issues related to rough sleeping.	To provide an approach to increase young people's knowledge on wider health issues and health literacy and strengthen their social interaction with service providers and peers to support community action.	To help practitioners to improve their knowledge and ability to promote health/oral health with people experiencing homelessness or at risk of becoming homeless.	To support practitioners and support workers with delivering training underpinned by evidence-based information on oral health	To provide useful information to improve the everyday life and well-being of people who are homeless.	To enhance sexual health among homeless people by discussing sexual and reproductive health and rights (SRHR)	To provide opportunities to improve the mental skills and strengths, wellbeing and social inclusion of young people experiencing homelessness or at risk.	To support students and practitioners from the health and social care sectors to be more sensitive and prepared to engage, and to discuss health promotion issues in a creative and meaningful way.
Summary of the findings	The co-design process successfully implemented principles of inclusion, equity, respect, capacity building, and purposefulness. Participants stated meaningful and valuable interventions.	Critical consciousness as an educational tool supported: 1. “trust building and collective engaging”. 2. “Constructing knowledge and developing skills provided to increase young people's knowledge, health literacy, and strengthen their social interaction”.	The preliminary findings show that lack of empathy from practitioners. Participants stated that continuity of care and stigma are barriers to accessing services.	N/Applicable	Findings show significance of considering the perspectives of both homeless individuals and service providers. Findings show the value of a living lab approach for addressing complex social issues like homelessness.	The “Snacka Sex” educational programme successfully provided homeless adults with knowledge and a safe space to discuss sexual health, rights, and norms.	Training programmes for self-regulation improve physical, mental, and social health and. Training programmes support positive transitions to independent living.	N/Applicable
Recommendations	Codesign needs sufficient resources and commitment. Need to involve people who are homeless and maintain their involvement.	Use Freire's educational approach as a framework to promote health and oral health for young people experiencing homelessness. Importance of interaction with the NGO settings.	N/Applicable	N/Applicable	Use the voices of people with lived experience and service providers to design and evaluate interventions. Use living lab as an approach to codesign.	Organisations should actively be involved in codesign as they recognize and advocate for service users.	Support the use of TIDieR checklist as a valuable framework for systematically describing MST4Life™.	N/Applicable

One study focussed on people sleeping rough (25), one study focussed on young people from 18 to 22 years old (9) and the other three studies (24, 26, 27) did not specify any age or any special circumstances of participants in the homelessness context. The types of educational / training materials developed from the five studies were diverse in nature and aims. The intervention from Mullins et al. was a three-pronged information strategy including an informal magazine, a website, and a dissemination event that developed a “Homelessness Protocol” with information to help those who are rough sleepers (25). A web app called “Ask Izzy”, containing information on services' in Australia was developed by Burrows et al. (24). Two studies developed educational programmes focusing on wider health promotion issues: Rodriguez et al. (9) co-designed a workshop programme exploring eight health and social participation topics (including oral health, mental health, healthy diet, drug abuse, resilience among others) and Wikström et al. (26) co-designed the development of a sex educational programme focused on three themes: (1). body and anatomy, (2). Sexuality, consent drugs and safer sex and (3). relations and relationships. One study co-developed a psychoeducational training program focused on mental health skills and wellbeing (27).

### 3.2 Methods and co-design approaches of included studies

The five studies had different co-design elements and phases: Semi-structured interviews (9, 24, 25) surveys, preparatory meetings with staff from the partners organisations and people with lived experience, and workshop sessions (9, 25, 26). Three studies presented information on elements/principles related to the co-design process they viewed as key (9, 24, 25). Mullins et al. highlighted inclusion as a core principle that should be aligned with the following elements: selecting appropriate group members; making participation a positive experience; and clarity of expectations at every stage of the research (25). Rodriguez et al. used critical dialogue, critical consciousness, and action for change from Critical Pedagogy in the co-design process (9). Burrows et al. choose the living lab approach, bringing together the different perspectives and capabilities from academia, industry, government, and citizens, to create the mobile app with a holistic view (24). Two studies (9, 24), two guides (26, 29) and one conference abstract (25) used the term co-design, and Burrows et al. (23) used the term co-creation to describe their approaches.

### 3.3 Barriers and enablers of co-designing health and oral health training/educational materials

Barriers and enablers in the co-design process to develop educational/training materials were identified and are presented in Table 3.

**Table 3:** Enablers and barriers in the co-design process to develop the educational/training materials.

Title	Enablers	Barriers
“No-one has listened to anything I’ve got to say before”: Co-design with people who are sleeping rough	• Selecting individuals based on commitment to attend research activities and diverse experiences of rough sleeping (Inclusivity). • Ensuring tangible benefits (reimbursements, meal vouchers, referrals for support services) • Promote a sense of belonging and value during activities. • Promote a safe environment and use group agreement outlining behavioural expectations. • Be flexible and promote informal interactions. • Monitoring participant's well-being during sessions • Clear communication and consent agreements.	• Difficulty in attracting marginalized groups for participating in research. • Difficulty in recruiting individuals under 25 years old. Natural attrition impacting the continuity of participants in the co-design process. Possible power differentials. Negative effect of COVID 19 • Lack of access to software or skills to participate in online meetings
Strengthening Social Interactions and Constructing New Oral Health and Health Knowledge: The Co-design, Implementation, and Evaluation of a Pedagogical Workshop Program with and for Homeless Young People	• Welcoming space by establishing a safe and non-judgmental environment. • Sharing meals, and informal chats before the workshops (including participants and research team). • Selecting key partners. • Using Critical Consciousness to explore sensitive topics and encourage critical reflection. • Good communication and flexibility from researchers. • Acknowledgement of participants’ previous knowledge.	• Sustainability.
Technology for societal change: Evaluating a mobile app addressing the emotional needs of people experiencing homelessness	• Using emotion-led approach. • Using of a living lab approach to involve various stakeholders. • Discussing realistic expectations of the service users.	• Maintaining momentum with the delivery of the web app. • Resources to sustain the process
Sexual and reproductive health and rights (SRHR) education with homeless people in Sweden	• Good engagement of participants. • Preparatory meetings to support the development of inclusive sessions. • Tailored to needs and desires of the participants. • Prioritising ethical aspects by not collecting detailed sociodemographic data increase participation. • The dual role of implementers and researchers provided deeper insights into the situation studied and allowed for active involvement in the change process.	• Terminologies and concepts. • Adapting to various accommodation lengths and community settings. • Challenges with financial and human resources associated with the constant adaptations needed.
Corrigendum to “The My Strengths Training for Life™ program: Rationale, logic model, and description of a strengths-based intervention for young people experiencing homelessness” [Evaluation and Program Planning 91 (2022) 102045]	• Collaborative research methodology. • Long-term successful partnership with stakeholders. • Sharing lessons learned for the benefit of policymakers and practitioners. • Flexibility and adaptation to needs and contexts. • Employing various formal and informal methods to engage stakeholders. • Embracing reflective practice	• Terminologies and concepts. • Adapting to various accommodation lengths and community settings. • Challenges with financial and human resources associated with the constant adaptations needed.

#### 3.3.1 Barriers

##### 3.3.1.1 Difficulty in recruiting, supporting and sustaining participation in the co-design process

Mullins et al. described difficulty in recruiting individuals that are perceived as marginalised, especially those individuals under the age of twenty-five (25). Mullins also described challenges during data collection due to lack of participants' previous experience in research such as the lack of access to software or skills to participate in online meetings (25), whilst Wikström et al. described literacy levels amongst participants impacting on ability to participate in reading and writing activity (26). Mullins et al. highlighted how participants' health issues or personal circumstances impacted their ability to continue to participate (25). Burrows et al. stated that one of the challenges was to sustaining participation and maintain the “momentum” after the delivery of the web app (24) as users had to return to the app after seven days via peer-to peer recommendation to feed into the evaluation process. The COVID-19 pandemic negatively impacted the dissemination phase of Mullins's output (25). The need to adapt the training program to various accommodation lengths and community settings presented a challenge for Cumming et al. (27).

##### 3.3.1.2 Power differentials

Mullins et al. identified power differentials as a challenge, e.g., participants becoming dismayed when their preferred idea was not deliverable due to the current systems in place beyond the control of the co-design process (25).

##### 3.3.1.3 Limited resources

For Wikström et al. the lack of continuity of certain activities due to limited funding was an issue (26). Cumming et al. (27) described the need for continuous evaluation and review of evolving needs of heterogeneous groups, demanding consistent effort and resource from the project.

#### 3.3.2 Enablers

##### 3.3.2.1 Inclusivity

Diverse and interconnected actions to ensure inclusivity of participants in different aspects of a co-design project were outlined. With regards to recruitment, identification of appropriate and established partners who already hold participants' trust and have an in depth knowledge of their life contexts resulted in effective methods to contact participants (9, 25). Reimbursement for participants' time e.g., meal vouchers, and referrals for support services to address diverse needs were offered as a way to increase participation and inclusion (25, 27). A gift pack to generate interest in one of the events was provided by Wikström et al. containing information about HIV and hepatitis, hepatitis vaccination cards and local sexual health services as well items of hygiene and safe sex (shower cream, body lotion, lubricants, condoms, and confectionary) (26).

During the initial design stages of studies, preparatory meetings with staff from the partners' organisations guided the development of tailored and inclusive sessions based on the needs of the participants, likely contributing to their positive feedback about the research (9, 26). To include people with writing and reading difficulties into the sessions, visual materials such as pictures and short films were used (26), as well as accessible language (25) and the use of different ways to facilitate self-expression such as games, drama, drawing, and collage were also offered (9).

##### 3.3.2.2 Safe environment for positive participation

Cummings et al. Mullins et al. and Wikström et al. set ground rules for and with participants by formulating a group agreement outlining behavioural expectations for a respectful interaction, such as showing respect for different opinions, and maintaining confidentiality about other participants' stories (25–27). Rodriguez et al. created a welcoming atmosphere by establishing a non-judgmental listening, creative, and pleasant environment which involved shared meals, and informal chats to build trust between participants and researchers before the activities (9). A safe environment was also reinforced by participant's well-being being monitored during sessions (25) through a deeper understanding of the needs and concerns of participants (24, 27). Good channels of communication between participants and researchers/facilitators led to participants feeling welcomed, safe, happy, committed, enthusiastic, and with a strong sense of belonging to the project (9, 25). Mullins et al. showcased that when working with people experiencing homelessness it is essential to show empathy, respect, and equal treatment (25). Trust building among participants and collective engagement were perceived as key elements that form a safe environment for positive and active participation (9). This is characterized by the existence of opportunities to have open discussions, with spontaneity and creativity, by hearing and sharing sensitive experiences, and life circumstances (9).

##### 3.3.2.3 Empowerment

Rodriguez et al. described empowerment of participants to have their voices heard and needs understood by those providing services, as well as changing unhealthy habits, as a positive outcome of participation (9). In addition, Mullins et al. and Burrows et al. reinforced how participation in those studies made participants feel their voices were heard and valued (24, 25). The acknowledgement of participants' previous knowledge and life experiences resulted in increased self-esteem, mutual learning process and the construction of new relationships between participants and their service providers (9). Hegemonic ideas about people experiencing homelessness as people with lack of motivation to engage with health services/practitioners might be linked with a paternalistic style of interaction adopted by professionals (a top-down approach, with just one way of communicating) that led to feelings of passivity and powerlessness for those marginalised groups using the services (9). Mullins et al. described how constant reinforcement of the project's goals and the participants roles led to empowerment and active participation (25).

Critical consciousness, formulated by Freire, is characterized by the depth and commitment of how individuals interpret current problems (9). Rodriguez et al. (9) stated that the critical reflexion about participants' life during the workshops, as part of critical consciousness, allowed the exploration of sensitive topics that encouraged participants to question structures of power in society. By doing this, participants felt confident to critically think about their status, identities, self-stigmatization, and responsibilities that leads to socio-political engagement for change (9). The impact of participating in co-design studies resulted in a range of opportunities for capacity building (25) such as the development of certain skills: active listening, health literacy, critical dialogue, and confidence to share their views about health-related issues (9). The opportunity to share similar stories helped participants to support others in the same situation (9, 25), and to make a collective agreement for behaviour change into health habits (9).

##### 3.3.2.4 Flexibility within the project

Flexibility from researchers in response to the needs of participants was an enabler for the co-design process (24, 25). Cummings et al. (27) highlighted that methodologies and models in research should respond to these needs and embrace reflective practice (27). The constant collection of participants' feedback during the process was perceived as important (9) enabling successful ongoing adjustments and appropriate changes being made in each phase of the study (26).

## 4 Discussion

Our findings suggest that components of the co-design process such as inclusivity, safe environment, empowerment, and flexibility can increase participation of people experiencing homelessness in research and in the development of educational materials. We have identified enablers to facilitate this process, the included studies demonstrated that stigmatised and vulnerable groups such as people experiencing homelessness, despite being perceived as “hard to reach” groups, are willing to take part in research if they felt included and could have their voices heard in a safe environment. A review by Ní Shé et al. (32) found that engagement with seldom-heard groups needs to occur in safe, accessible, and inclusive spaces. Therefore, importance of providing an emotionally safe environment for positive participation based on principles of respect, non-judgmental listening, with meaningful opportunities for participants to feel that their views and lived experience have been acknowledged is required.

In our review, participants' feelings of being safe to express themselves within the research environment resulted in a feeling of empowerment, leaving them confident to share their views on issues that were important to them. There are other studies that reinforce the links between the provision of a safe environment and the empowerment of participants as enablers for participation when mutual trust, equity, and empathy are embedded in all phases of the research process. Schiffler et al. (33) identified clients were reportedly empowered to achieve their personal goals when co-designed mental health interventions were provided in their living environment, including home, work, and other places that they identified as safe and favourable. Flexibility was perceived as a key element to be applied across the different research's stages as an important strategy to involve people who might otherwise be excluded of participating. Life crisis and financial issues can be challenges for participation. The findings of our review suggests that incentives are an enabler in the codesign process, which concurs with the review finding by Ní Shé et al. (32) where necessary costing and flexibility in payment should be included when designing research with vulnerable groups. Flexibility related to researcher's attitude of being sensitive to participants' feedback and expressed needs during the process resulted in positive changes on research activities (time, duration, ways of delivering). Therefore, the context and needs of people experiencing homelessness are complex and diverse and research processes with less rigid structures can better allow the accommodation of necessary changes.

There were benefits in using co-design identified from the review. The included study by Rodriguez et al. reported impact from the co-design process with reported improvement in individual's critical consciousness, health literacy and behaviour change (9). It also helped strengthen their social interaction with service providers and their peers towards a more critical involvement with their communities. Social justice to achieve health equity should be core practices for health promotion interventions. Participants felt empowered when conditions for active involvement are in place and when they receive equitable treatment. These elements are essential to undoing oppressive forces existing in power structures (5, 34–36). Tindall et al. identified that co-design was helpful in balancing the power differential and providing support when participants usually feel reduction in their power especially in mental health settings where there are inherent power imbalances (37).

Health promotion interventions using participatory research methods such as co-design are successful because they consider the context and the specific needs of target audiences (38). Three of the included studies highlighted how important it is to have an in depth understanding of the context and needs of participants in order to tailor the research activities to enable participation (9, 25, 27). This led to empowerment of participants that felt more equipped to take informed decisions and change towards a healthier life. Health promotion is a process that enables people to increase control over and improve their health (39). Knowledge exchange programmes with public engagement activities have recommended the involvement of young people experiencing homelessness in the co-design of training resources to be used by practitioners (7). Adding to this, the participation of socially excluded groups, such as families, children and young people experiencing poverty and homelessness, using co-design approaches have benefited from the construction of new oral health and health knowledge (9, 40). Therefore, an alternative approach is necessary to empower people, enabling their active participation and to take charge of their own lives and environments (41).

The perceived barriers to codesign in research of increased time and financial expenditure are corroborated by Slattery et al. (18) e.g., there is not enough time allocated or enough focus on development of the skills needed to build trust and long-term partnerships within the community.

### 4.1 Research gap

This review identified substantial gaps in the literature. Only five studies used co-design methods in the development of health and/or oral health educational/training materials with people experiencing homelessness and/or their support workers. We suggested that limited time and resources to conduct research with co-design elements are key factors for the limited evidence. The provision of inclusive resources that ensure wider participation of people experiencing homelessness from the recruitment to dissemination phases is challenging and requires constant training, reflexive practice, and skills “development from researchers”. The use of reporting frameworks relevant to study design in the existing literature is limited and reduces the ability to identify all the active components in the co-design process, future studies in this area should utilise study design appropriate reporting frameworks.

### 4.2 Strengths and limitations

To the best of the authors' knowledge this is the first review to bring together and examine research on co-design of oral health and health resources with participation of people with lived experience in homelessness. Two long-term partner organisations working in the homelessness sector reviewed the first draft of this manuscript and made their comments. The use of JBI methods to inform the review, registration of protocol, extensive search strategy and contact with a substantial number of national and international stakeholders' experts in the field were the key strengths of our review. A Quality Appraisal of the included studies, although not a requirement for scoping reviews was completed, providing a greater sense of the overall quality of existing research in this field. A limitation of the search strategy was our focus on English language only publications.

## 5 Conclusion

The evidence in this area is limited. This review provides foundations for further research to examine the impact of different components of co-design including the environment in which the co-design process is conducted. The identified enablers to co-design health and/or oral health educational/training materials suggest that an active and positive engagement with participants promotes meaningful experience of participation, resulting in participants' empowerment and increased knowledge. An in-depth knowledge of the diverse contexts and views of people experiencing homelessness through the investment of time and creation of good channels of communication, trust and positive interaction enables their voices to be heard, validated, and used to develop resources that can help practitioners with the non-stigmatisation of these groups in healthcare settings and society. Training or educational programmes/materials that include the views of people with lived experience of the health issues to be addressed have an increased chance of success in to improving service users' lives and wellbeing. Future endeavours should foster increased collaboration with individuals with lived experience of homelessness to co-design health and oral health promotion training/educational materials. Further studies with experimental design and reported using appropriate study design frameworks detailing active components of the co-design process would strengthen the evidence base in this area.

## Data Availability

The raw data supporting the conclusions of this article will be made available by the authors, without undue reservation. The protocol for this review was registered with the Open Science Framework and can be accessed from the following web page: https://osf.io/7hbac.

## References

[1] Liu M Hwang SW. Health care for homeless people. Nat Rev Dis Primers. (2021) 7(1):5. 10.1038/s41572-020-00241-2 33446661

[2] Legalisation. Housing (Scotland) Act 1987. Available online at: https://www.legislation.gov.uk/ukpga/1987/26/contents (Accessed December 11, 2023).

[3] Somerville P. Understanding homelessness. Housing. (2013) 30:1. 10.1080/14036096.2012.756096

[4] Xiao S Snider C Pinto A Handford C. Co-designing with communities to evaluate an ED outreach worker program for people experiencing homelessness: protocol and preliminary findings of a community-based participatory research study. Int J Integr Care. (2022) 22:1–2. 10.5334/ijic.icic22212

[5] Chandanabhumma PP Narasimhan S. Towards health equity and social justice: an applied framework of decolonization in health promotion. Health Promot Int. (2020) 35(4):831–40. 10.1093/heapro/daz053 31236575

[6] Beaton L Humphris G Rodriguez A Freeman R. Community-based oral health interventions for people experiencing homelessness: a scoping review. Community Dent Health. (2020) 37(2):150–60. 10.1922/CDH_00014Beaton11 32212437

[7] Rodriguez A Dalcin CB Fernandes F Freeman R Humphris G. Helping Young People Feel at Home in Scotland: Building Collaborative and Integrated Services for Youth Homeless: A Reflexive Mapping Approach for Health and Social Care Integration. Dundee, United Kingdom: University of Dundee (2020).

[8] Doughty J Macdonald ME Muirhead V Freeman R. Oral health-related stigma: describing and defining a ubiquitous phenomenon. Community Dent Oral Epidemiol. (2023) 51(6):1078–83. 10.1111/cdoe.12893 37462247

[9] Rodriguez A Beaton L Freeman R. Strengthening social interactions and constructing new oral health and health knowledge: the co-design, implementation and evaluation of a pedagogical workshop program with and for homeless young people. Dent J. (2019) 7(1):11. 10.3390/dj7010011 PMC6473716 30717131

[10] Sanders E. From User-Centered to Participatory Design Approaches. United Kingdom: Taylor & Francis Books Limited (2002). p. 1–7.

[11] Moll S Wyndham-West M Mulvale G Park S Buettgen A Phoenix M Are you really doing ‘codesign’? Critical reflections when working with vulnerable populations. BMJ open. (2020) 10(11):e038339. 10.1136/bmjopen-2020-038339 33148733 PMC7640510

[12] Sartor C Sunkel C. Perspectives: involving persons with lived experience of mental health conditions in service delivery, development and leadership. BJPsych Bull. (2022) 46(3):160–4. 10.1192/bjb.2021.51 33977895 PMC9346508

[13] McHugh N Baker R Bambra C. Policy actors’ perceptions of public participation to tackle health inequalities in Scotland: a paradox? Int J Equity Health. (2023) 22(1):57. 10.1186/s12939-023-01869-8 36997962 PMC10062251

[14] Organization WH. WHO Framework for Meaningful Engagement of People Living with Noncommunicable Diseases, and Mental Health and Neurological Conditions. World Health Organisation (2023).

[15] Sanders EB-N. From user-centered to participatory design approaches. In: Frascara J, editor. Design and the Social Sciences. CRC Press (2002). p. 18–25.

[16] Visser FS Stappers PJ Van der Lugt R Sanders EB. Contextmapping: experiences from practice. CoDesign. (2005) 1(2):119–49. 10.1080/15710880500135987

[17] Hussey D Trinder-Widdess Z Dee C Bagnall D Bojangles T Kesten JM. Co-design of harm reduction materials for people who inject drugs to implement research findings. Harm Reduct J. (2019) 16:1–11. 10.1186/s12954-019-0300-z 31174536 PMC6555749

[18] Slattery P Saeri AK Bragge P. Research co-design in health: a rapid overview of reviews. Health Res Policy Syst. (2020) 18(1):17. 10.1186/s12961-020-0528-9 32046728 PMC7014755

[19] Peters MD Marnie C Tricco AC Pollock D Munn Z Alexander L Updated methodological guidance for the conduct of scoping reviews. JBI Evid Implement. (2021) 19(1):3–10. 10.1097/XEB.0000000000000277 33570328

[20] Tricco AC Lillie E Zarin W O'Brien KK Colquhoun H Levac D PRISMA Extension for scoping reviews (PRISMA-ScR): checklist and explanation. Ann Intern Med. (2018) 169(7):467–73. 10.7326/M18-0850 30178033

[21] Ouzzani M Hammady H Fedorowicz Z Elmagarmid A. Rayyan—a web and mobile app for systematic reviews. Syst Rev. (2016) 5:1–10. 10.1186/s13643-016-0384-4 27919275 PMC5139140

[22] Lockwood C Munn Z Porritt K. Qualitative research synthesis: methodological guidance for systematic reviewers utilizing meta-aggregation. JBI Evid Implement. (2015) 13(3):179–87. 10.1097/XEB.0000000000000062 26262565

[23] Hong QN Pluye P Fàbregues S Bartlett G Boardman F Cargo M Mixed methods appraisal tool (MMAT), version 2018. Reg Copr. (2018) 1148552(10):3. 10.3233/EFI-180221

[24] Burrows R Mendoza A Pedell S Sterling L Miller T Lopez-Lorca A. Technology for societal change: evaluating a mobile app addressing the emotional needs of people experiencing homelessness. Health Informatics J. (2022) 28(4):14604582221146720. 10.1177/14604582221146720 36548199

[25] Mullins RM Kelly BE Chiappalone PS Lewis VJ. ‘No-one has listened to anything I’ve got to say before’: co-design with people who are sleeping rough. Health Expect. (2021) 24(3):930–9. 10.1111/hex.13235 33756006 PMC8235881

[26] Wikström E Eriksson E-M Lindroth M. Sexual and reproductive health and rights (SRHR) education with homeless people in Sweden. Sex Educ. (2018) 18(6):611–25. 10.1080/14681811.2018.1451320

[27] Cumming J Clarke FJ Holland MJ Parry BJ Quinton ML Cooley SJ. A feasibility study of the my strengths training for Life™MST4Life™) program for young people experiencing homelessness. Int J Environ Res Public Health. (2022) 19(6):3320. 10.3390/ijerph19063320 35329014 PMC8950686

[28] Braun V Clarke V. Using thematic analysis in psychology. Qual Res Psychol. (2006) 3(2):77–101. 10.1191/1478088706qp063oa

[29] Rodriguez A Biazus Dalcin C McGoldrick N van Blerk L Murray C Freeman R. Co-designing a training package to promote health/oral health for people experiencing homelessness. Eur J Public Health. (2021) 31(3):ckab164.364. 10.1093/eurpub/ckab164.364

[30] Rodriguez A Biazus-Dalcin C Marshall J Gorman R. Smile4life: A Co-Designed Educational and Training Resource Guide. Dundee, United Kingdom: NHS Education for Scotland (2022).

[31] Rodriguez A Biazus-Dalcin C van Blerk L. ‘Do Not Give Up On Us’: A Workshop Guide for Health Promotion and Civic Engagement. Dundee, United Kingdom: University of Dundee (2022).

[32] Shé ÉN Morton S Lambert V Cheallaigh CN Lacey V Dunn E Clarifying the mechanisms and resources that enable the reciprocal involvement of seldom heard groups in health and social care research: a collaborative rapid realist review process. Health Expect. (2019) 22(3):298–306. 10.1111/hex.12865 30729621 PMC6543157

[33] Schiffler T Kapan A Gansterer A Pass T Lehner L Gil-Salmeron A Characteristics and effectiveness of co-designed mental health interventions in primary care for people experiencing homelessness: a systematic review. Int J Environ Res Public Health. (2023) 20(1):892. 10.3390/ijerph20010892 36613214 PMC9820061

[34] Freire P. Pedagogy of the Oppressed. New York, USA: New York Seabury Press (1970).

[35] Nutbeam D. Evaluating health promotion—progress, problems and solutions. Health Promot Int. (1998) 13(1):27–44. 10.1093/heapro/13.1.27

[36] Koh HK Oppenheimer SC Massin-Short SB Emmons KM Geller AC Viswanath K. Translating research evidence into practice to reduce health disparities: a social determinants approach. Am J Public Health. (2010) 100(Suppl 1):S72–80. 10.2105/AJPH.2009.167353 20147686 PMC2837437

[37] Tindall RM Ferris M Townsend M Boschert G Moylan S. A first-hand experience of co-design in mental health service design: opportunities, challenges, and lessons. Int J Ment Health Nurs. (2021) 30(6):1693–702. 10.1111/inm.12925 34390117

[38] Scott DAH Currie C Stones T Scott C John J Wanyonyi K. Co-design of an oral health promotion animated film with families in the South of England. Br Dent J. (2020) 228(3):164–70. 10.1038/s41415-020-1208-4 32060458

[39] World Health Organization. The Ottawa Charter for Health Promotion Geneva1986. Available online at: http://www.who.int/healthpromotion/conferences/previous/ottawa/en/ (Accessed December 11, 2023).

[40] Nanjappa S Freeman R. CHATTERBOX: developing and piloting an interactive communication toolkit for engaging families with dental services. J Nurs Care. (2014) 3:3–6. 10.4172/2167-1168.1000215

[41] Zamenopoulos T Alexiou K. Co-Design as Collaborative Research: Bristol University/AHRC Connected Communities Programme. United Kingdom: University of Bristol/AHRC Connected Communities Programme (2018).

