# Supplementary material for: Co-design of health educational materials with people experiencing homelessness and support workers: a scoping review
Source: Front Oral Health. 2024 Jun 11;5:1355349. doi: 10.3389/froh.2024.1355349 (PMC11196637; doi:10.3389/froh.2024.1355349)
Supplement: Supplementary file 1 [file Datasheet1.docx]

Supplementary Appendix 1. General database search terms.

| String 1 Homeless  Truncation* | Homeless* |
| --- | --- |
| String 2 Homeless specific  Truncation* | Homeless Persons OR Homeless Youth OR Homeless AND (group* OR population OR people OR adult* OR person* OR youth OR young) OR Runaway* OR Rough sleeper* OR "Sleeping rough" |
| String 3 Support worker  Truncation* | Provider* OR Caregiver* OR Practitioner* OR Staff OR Support OR Professional* OR Worker* |
| String 4 Health promotion  Truncation* | Oral Health OR Dental Care OR Health* OR Health promotion |
| String 5 Co-design  Truncation* | Co-design OR Co-creat* OR Co-working OR Co-participation |
| String 6 Training  Truncation* | Training OR Education* OR Information OR Workshop* OR Material* |
|  | Combine String 1 AND String (2 OR 3) AND String 4 AND String 5 and String 6 |
