# Supplementary material for: Co-design of health educational materials with people experiencing homelessness and support workers: a scoping review
Source: Front Oral Health. 2024 Jun 11;5:1355349. doi: 10.3389/froh.2024.1355349 (PMC11196637; doi:10.3389/froh.2024.1355349)
Supplement: Supplementary file 2 [file Datasheet2.docx]

Supplementary Appendix 2- Quality assessment of the included studies results

| Study | Quality | Quality Appraisal tool |
| --- | --- | --- |
| Mullins et al. [27] | Medium | JBI Critical Appraisal Checklist for Qualitative Research (22) |
| Burrows et al. [25] | High | MMAT Mixed Methods Appraisal Tool (23) |
| Wikström et al. [28] | Medium | JBI Critical Appraisal Checklist for Qualitative Research (22) |
| Cumming et al. [26] | Low | JBI Critical Appraisal Checklist for Qualitative Research (22) |
| Rodriguez et al. [9] | High | JBI Critical Appraisal Checklist for Qualitative Research (22) |
